# The *Schistosoma* Granuloma: Friend or Foe?

**DOI:** 10.3389/fimmu.2013.00089

**Published:** 2013-04-15

**Authors:** Emily Hams, Gabriella Aviello, Padraic G. Fallon

**Affiliations:** ^1^Trinity Biomedical Sciences Institute, School of Medicine, Trinity College DublinDublin, Ireland; ^2^National Children’s Research Centre, Our Lady’s Children’s HospitalDublin, Ireland; ^3^Institute of Molecular Medicine, St James’s HospitalDublin, Ireland

**Keywords:** *Schistosoma mansoni*, granuloma, inflammation, fibrosis, immunology

## Abstract

Infection of man with *Schistosoma* species of trematode parasite causes marked chronic morbidity. Individuals that become infected with Schistosomes may develop a spectrum of pathology ranging from mild cercarial dermatitis to severe tissue inflammation, in particular within the liver and intestines, which can lead to life threatening hepatosplenomegaly. It is well established that the etiopathology during schistosomiasis is primarily due to an excessive or unregulated inflammatory response to the parasite, in particular to eggs that become trapped in various tissue. The eggs forms the *foci* of a classical type 2 granulomatous inflammation, characterized by an eosinophil-rich, CD4^+^ T helper (Th) 2 cell dominated infiltrate with additional infiltration of alternatively activated macrophages (M2). Indeed the sequela of the type 2 perioval granuloma is marked fibroblast infiltration and development of fibrosis. Paradoxically, while the granuloma is the cause of pathology it also can afford some protection, whereby the granuloma minimizes collateral tissue damage in the liver and intestines. Furthermore, the parasite is exquisitely reliant on the host to mount a granulomatous reaction to the eggs as this inflammatory response facilitates the successful excretion of the eggs from the host. In this focused review we will address the conundrum of the *S. mansoni* granuloma acting as both friend and foe in inflammation during infection.

Schistosomiasis is a major chronic disease of humans in endemic regions. The schistosome species of major medical relevance to man are *Schistosoma mansoni*, *S. haematobium*, and *S. japonicum*. In this review we will focus on granulomatous inflammation following *S. mansoni* infection. Whilst in the majority of cases people infected with *S. mansoni* are relatively asymptomatic, or show restricted morbidity associated with intestinal inflammation and fibrosis, a minority of infected individuals develop a severe hepatosplenic schistosomiasis (HS). HS is characterized by hepatic fibrosis, hepatosplenomegaly, and portal hypertension, and can result in death in the absence of medical attention. A central feature of the pathology associated with *S. mansoni* infection is the development of granulomatous inflammation around parasite eggs that become trapped in tissue, in particular the liver and intestines. The host’s immune response generated against parasite antigens plays a critical role in both dictating the severity of tissue inflammation and associated disease. Paradoxically, the host immune response to the parasite also facilitates parasite replication and survival. This review will focus on the immunobiology of the egg-associated granuloma elicited during *S. mansoni* infection and will address the conundrum of the *Schistosoma* granuloma eliciting inflammation that acts as both friend and foe during infection.

## The Immunopathogenesis of *Schistosoma mansoni*

Humans become infected with *S. mansoni* following exposure to water contaminated with skin penetrating cercariae. The *S. mansoni* cercariae are highly motile organisms able to enter the host *via* the penetration of intact skin. The cercariae transform into schistosomula after entering the skin and migrate *via* the vasculature and lymphatics through the lungs to the hepatic portal system. Schistosomula differentiate to male and female schistosomes, pair, and migrate to the mesenteric venous plexus, where adult worms can live for 5–10 years. Female schistosomes produce ∼300 eggs each day that are laid in mesenteric circulation. The eggs are viable, metabolically active organisms, and are highly antigenic. *S. mansoni* eggs adhere to the endothelium of mesenteric blood vessels and evoke inflammation leading to a granulomatous response that is necessary for translocation into the intestinal lumen and excretion in the feces (DeFranco et al., 2007). Indeed eggs have been shown to preferentially enter the Peyer’s patches within the intestinal wall to facilitate egress of eggs to the intestinal lumen (Turner et al., 2013). Eggs that pass through the intestinal wall are excreted in the feces and if deposited in fresh water, may infect an appropriate species of snail, thus propagating the life cycle. However, some of the eggs may also become lodged in the host’s intestine, liver, or other sites, where they can cause the morbidity and mortality associated with schistosomiasis *mansoni*.

Clinical signs of schistosomiasis are dependent on the maturation stage of parasites and their eggs. In humans, acute infection is characterized by a debilitating febrile illness (Katayama fever) that usually occurs before the appearance of eggs in the stool, having a peak 6–8 weeks after infection. In chronic disease, eggs trapped in various tissues evoke the formation of granulomatous inflammation, which along with the ensuing fibrosis cause the majority of pathological conditions. In individuals that develop HS, liver portal tract fibrosis leads to obstructive vascular lesions, portal hypertension, ascites, and fatal bleeding from esophagogastric varices. Collectively, granulomatous inflammation around parasite eggs is a cardinal feature of schistosomiasis *mansoni* and the egg-associated pathology is central to the morbidity and indeed mortality that occurs in infected humans.

The use of animal models has facilitated the advancement of our understanding of the immunopathology during *S. mansoni* infection, with the mouse the most widely used species. It must be stressed that *S. mansoni* infection of mice does not faithfully recapitulate all aspects of human schistosomiasis (Fallon, 2000). Crucially, mice do not appear to develop portal tract fibrosis (Symmers’ pipe stem fibrosis) that is associated with morbidity in humans, instead pathology in mice is primarily associated with a granulomatous responses to parasite eggs trapped in the host tissue, primarily in the liver and intestines. Recently, an interesting caveat to the use of inbred laboratory mouse strains was shown, with *S. mansoni* infection of wild outbred mice leading to more marked disease than inbred strains. The observed increased pathology in outbred animals was specifically associated with interleukin (IL)-1 elicited IL-17 producing CD4^+^ T helper (Th)17 cells (Smith et al., 2011). Nevertheless mouse studies, in particular, the recent use of gene knockout or transgenic animals, has made fundamental advances in understanding the mechanisms of immunopathology of schistosomiasis.

Following infection of mice in the first 3–5 weeks, during which the host is exposed to migrating immature parasites, there is immune activation with a marked type 1 immune response, with increased Th1 cells and release of IL-12 and interferon (IFN)-γ. While the immune response during the initial weeks of infection with *S. mansoni* is strongly type 1-mediated, and primarily targeted against the worm antigens, it should be noted that type 2 responses are also primed. As the parasites mature, mate, and begin to produce eggs after 5–6 weeks, the immune response alters markedly alters, leading to a decrease in the type 1 immune component and concomitant emergence of a potent type 2 response (Pearce and MacDonald, 2002). The switch to a type 2-mediated response from 5 to 6 weeks post infection is a consequence of egg-production by mature female worms. *S. mansoni* eggs are potent inducers of type 2 responses when injected into naive mice (Vella et al., 1992). Furthermore, soluble egg-antigens (SEA) or antigenic egg secretions also induced a marked type 2 response. The egg-antigen stimulated type 2 response leads to Th2 cell expansion, production of IL-4, IL-5, and IL-13 accompanied by an upregulation in immunoglobulin (Ig) E levels and circulating eosinophils. The peak of this type 2 response corresponds with the maximal cell response against the egg and is closely associated with the magnitude of granulomatous inflammation surrounding the egg. During the chronic phase of infection, after ∼3 months, there is a marked decrease in the magnitude of the Th2 response and a state of hyporesponsiveness emerges. The potential for the Th2 response to lead to controlled chronic disease is part of a dynamic association between Th1, Th17, and T regulatory cells regulating disease severity during *S. mansoni* infection. For example, intestinal-associated CD4^+^CD25^+^FoxP3^+^T_regs_, which expand during chronic, schistosome-induced colitic inflammation, are capable of modulating Th2 *via* IL-4 suppression (Turner et al., 2011). More relevantly, a similar Th/reg cytokine interplay may also occur in man (Mbow et al., 2013).

## Immune Dependence of Granuloma Formation

**Figure 1:**
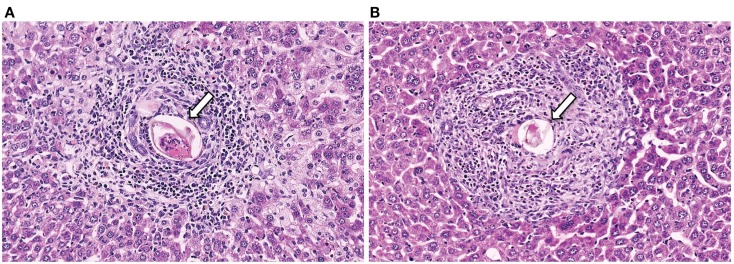
**Representative histology sections (stained with H&E) of livers from a CD4^+^ T cell depleted (A) and normal (B) mouse, with arrows to indicate the *S. mansoni* egg**.

The classic phenomenon associated with *S. mansoni* infection is the formation of multi-cellular granulomatous inflammation surrounding eggs trapped in various tissues (Figure 1). The granulomatous response to the egg is primarily orchestrated by CD4^+^ T cells. However, CD8^+^ T cells, B cells, and M2 macrophages have also been shown to play a role in regulating granuloma formation (Fallon et al., 1998; Jankovic et al., 1998; Herbert et al., 2004). In addition, eosinophils also form a prominent constituent of the granuloma (Moore et al., 1977). However, while marked eosinophil infiltration is a cardinal feature of the *Schistosoma* granuloma (Lenzi et al., 1987), the actual function of eosinophils in the granuloma is not known. The generation of tissue eosinophilia during *Schistosoma* infection of mice is mediated by type 2 cytokines, such as IL-5 and IL-13, (Sher et al., 1990; Chiaramonte et al., 1999; Fallon et al., 2000; Reiman et al., 2006). However, using two transgenic mouse strains deficient in eosinophils, there was no marked defect in worm burden, granuloma formation, and liver fibrosis following *S. mansoni* infection (Swartz et al., 2006). Further work is required to fully elucidate the functions of eosinophils within the *Schistosoma* granuloma. Similarly, while mast cells are also present within the *Schistosoma* granuloma (Weinstock and Boros, 1983; Lenzi et al., 1987), the actual involvement of such cells in the formation of the granuloma around the egg and subsequent resolution is not known.

The importance of T cells in the generation of the granuloma was initially shown in nude mice and animals subjected to T cell depletion; with T cell deficient mice having impaired granuloma formation around eggs (Byram and von Lichtenberg, 1977; Doenhoff et al., 1981). A dominant role for CD4^+^ T cells in granuloma formation was shown using depleting monoclonal antibodies (Mathew and Boros, 1986; Fallon et al., 1998). While it could be anticipated that the attenuated granuloma surrounding the egg in CD4^+^ T cell-deficient mice would lead to less pathology, the opposite occurred. Indeed there is striking mortality in *S. mansoni*-infected mice with a compromised immune system (Table 1). This highlights the paradox: the granuloma that forms to encapsulates the egg can lead to pathology but the granuloma also functions to protect the host.

**Table 1:** **Summary of the consequence of *S. mansoni* egg infection in immunologically intact and immune compromised (e.g., CD4^+^ T cell depleted) mice**.

	Liver				Intestine			Systemic			
	Gr^a^	Eo^b^	Fibrosis	Hepatocyte damage	Egg excretion	Eo^b^	Pathology	Antibody responses	T cells	Endotoxemia	Mortalities^c^ (%)
Immunologically intact mice	+	+	+	−	+	+	−	+	Th2/Treg	−	<5
Immune compromised mice	±	−	±	+	−	−	+	±	Th1/17	+	100

*^a^Gr, granulomatous inflammation*.

*^b^Eo, tissue eosinophilia*.

*^c^Mortalities are expressed as percentage (ranges) of mice dead by 56 days after an acute infection*.

**Figure 2:**
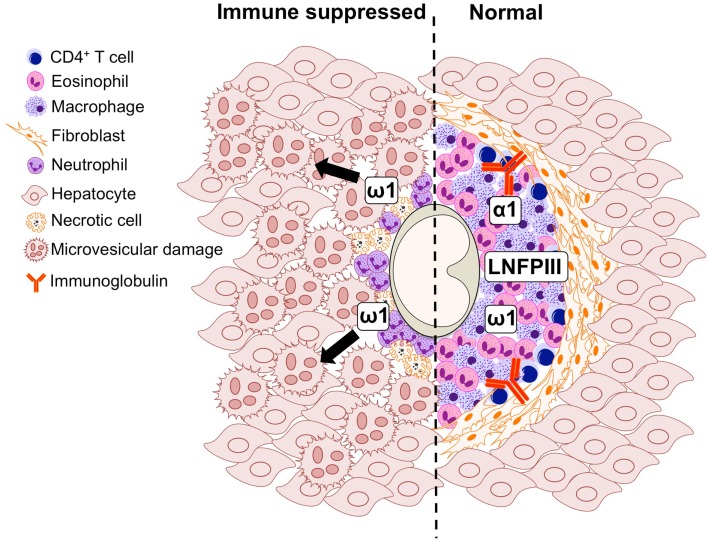
**Graphical representation of the cell populations involved in the formation of the *S. mansoni* egg-induced liver granuloma from an immune suppressed (*left*) and immunologically intact mouse (*right*)**.

**Figure 3:**
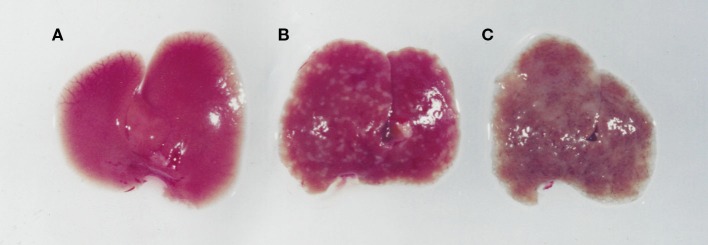
**Images of livers from an uninfected (A), an *S. mansoni*-infected immune competent (B), and immune suppressed (C) mouse**. While an infected immunologically intact mice has granulomas in the liver **(B)**, in an immunodeficient mouse **(C)** there is marked whitening of the liver due to microvesicular hepatosteatosis.

*S. mansoni* infection of mice with CD4^+^ T cells depleted develop an acute fatal disease, with animals dying from weeks 4–6 after infection; coincident with egg deposition in tissue (Fallon et al., 1998). In immunologically intact mice, the eggs that are deposited in the liver are encapsulated within the granuloma with hepatocytes outside the granuloma are overtly normal (Figure 1), with such mice having normal liver function. In contrast, in the absence of CD4^+^ T cells there is a limited granulomatous response, with the cellular infiltrate around the egg being neutrophil dominated as opposed to the eosinophil-rich granuloma observed in immunologically intact mice (Figure 2). Furthermore, without an intact functional granuloma there is extensive microvesicular damage to hepatocytes, and a consequential elevation in serum transaminase levels consistent with hepatocyte damage. Grossly, the hepatic steatosis is evident with the fat-laden white appearance of the liver in immune suppressed infected mice (Figure 3).

Therefore formation of a granuloma around the egg can be perceived as functioning to sequester egg secretions that can cause hepatotoxicity (Dunne et al., 1991). In addition to liver specific pathology, the absence of a functional granuloma in immune suppressed mice also leads to an inability to efficiently excrete eggs in feces, and consequentially eggs are trapped in the intestines leading to inflammation. Indeed this phenomenon of immune dependence of egg excretion (Doenhoff et al., 1981), illustrates the novel usurping of immunity by the parasite whereby the egg granuloma functions to induce a specific host immune response resulting in the translocation of the egg through the intestinal wall to be excreted in the feces. It is noteworthy that in other chronic granulomatous diseases, specifically tuberculosis, it is suggested that instead of limiting bacterial proliferation, the granuloma may actually benefit the bacteria (Ramakrishnan, 2012).

Crucially, these experimental observations on a role for CD4^+^ T cells in facilitating egg excretion in *S. mansoni*-infected mice also occur in humans. Karanja et al. examined egg excretion, i.e., detection of eggs in the feces, in a cohort of *S. mansoni*-infected individuals in Kenya that were seronegative or seropositive for human immunodeficiency virus (HIV). There was a positive association between egg excretion rates and levels of circulating CD4 in HIV^+^ patients, inferring a functional immune response was needed for egg excretion in man (Karanja et al., 1997). This may be *S. mansoni* specific phenomenon, or related to infection intensity, as in another study in Zimbabwe there was no such association between immune status and egg excretion in patients that were predominately infected with *S. haematobium* (Kallestrup et al., 2005).

The earlier studies in mouse models established an essential role for CD4^+^ T cells in granuloma formation. More recently, the role of Th1 cellular cytokines [such as Tumor Necrosis Factor (TNF)-α, IFN-γ, and IL-2], Th2 (such as IL-4, IL-5, IL-9, and IL-13), Th17 (IL-17), and T regulatory cells in granuloma formation have been elucidated (Singh et al., 2005; Rutitzky and Stadecker, 2011). For example, mice deficient in type 1 cytokines such as IFNγ and IL-12p40 show little alteration in pathology, whilst mice deficient in certain type 2 cytokines are unable to generate a granuloma and may develop exacerbated pathology (Wynn and Cheever, 1995). Thus IL-4 deficient mice have impaired granuloma formation and develop acute fatal cachexia (Brunet et al., 1997). Indeed in mice that are tolerized to egg-antigens, a type 1 biased response is evoked leading to hepatotoxicity and death (Fallon and Dunne, 1999). Furthermore, in the absence of IL-4 alone, or both IL-4 and IL-13, there is acute mortality with impaired egg excretion leading to endotoxemia (Fallon et al., 2000). Thus the generation of a functional granuloma is essential for a balanced cellular immune response ensuring survival of mice during infection with *S. mansoni*.

While a major focus has been on facets of adaptive immunity contributing to the formation of the egg granuloma, in recent years there is an increasing recognition that innate immunity also contributes. The generation of type 2 immunity by schistosome eggs requires antigen-presenting cells, such as dendritic cells (DC), processing and presenting schistosome egg-antigens (MacDonald et al., 2002). Indeed, depleting CD11c^+^ DCs during active *S. mansoni* infection severely impairs Th2 responses, suggesting that DCs are critical for Th2 induction (Phythian-Adams et al., 2010). DCs are equipped with an array of pattern recognition receptors (PRRs), including C-type lectin receptors (CLRs) and Toll-like receptors (TLRs), in order to recognize and differentiate between pathogens by binding pathogen-associated molecular patterns (PAMPs) and instruct the immune system to mount a dedicated response. In order to induce a Th2 response, SEA interferes with TLR-mediated DC activation (Kane et al., 2004). SEA can suppress maturation and cytokine production of human and murine DCs induced by activation with the TLR4 ligand LPS and the TLR3 ligand poly-I:C (MacDonald et al., 2002; Kane et al., 2004; van Liempt et al., 2007).

More recently, it has been shown that egg-antigens can also activate the NLRP3 inflammasome, in combination with a TLR agonist, leading to a release of IL-1β (Ritter et al., 2010). Such inflammasome activation modulates the immune response during *Schistosoma* infection, with mice deficient in NLRP3 developing smaller and more fibrotic granulomas (Ritter et al., 2010). In active infection it is feasible to conclude that TLR agonists such as LPS may leak through the intestinal wall and associate with egg-antigens to co-activate the inflammasome, leading to the release of IL-1β, which is essential for schistosome-related immunomodulation (Guo et al., 2009; Ritter et al., 2010). In this context it is relevant that in mice strains that develop more severe disease during *S. mansoni* infection, IL-1 receptor-associated kinase-like 2 (IRAK-2) was identified as a novel regulator of IL-1-induced pathogenic Th17 cells in schistosomiasis (Smith et al., 2011). The emergence of the importance of the inflammasome in the generation of granulomatous inflammation highlights the vital role for the innate immune response to the egg in generating the granuloma.

## The Egg Granuloma and Genesis of Fibrosis

A characteristic of *S. mansoni* infection is the development of fibrosis within the portal tracts of man and in mice within the egg granuloma. This pro-fibrotic property of the *S. mansoni* granuloma was used to identify that IL-13 was the dominant Th2 cytokine responsible for the development of liver fibrosis. While hepatic fibrosis is impaired in *S. mansoni*-infected mice unable to signal through IL-4Rα (*Il-4r*α^−/−^), it is ablated in mice treated with soluble IL-13Rα2-Fc and also fails to develop in mice deficient in IL-13 (*Il-13*^−/−^) (Chiaramonte et al., 1999; Jankovic et al., 1999; Fallon et al., 2000). In addition, *in vitro* studies have demonstrated the ability of IL-13 to directly stimulate collagen production in fibroblasts (Chiaramonte et al., 1999). The fibrogenic role of IL-13 involves the cytokine, together with IL-4, inducing the expression of arginase in macrophages *via* M2 polarization. Arginase uses l-arginine as a substrate to make l-ornithine, which is converted by ornithine aminotransferase to proline, a crucial amino acid for the production of collagen and the development of fibrosis (Hesse et al., 2001). The major function of Arg-1 is to downmodulate granulomatous inflammation in the liver and intestine and to slow the progression of Th2-dependent fibrosis in chronically infected mice (Pesce et al., 2009). The high expression of Arg-1, Ym-1, and FIZZ1 in granulomatous tissue reflects the large population of M2 macrophages and fibroblasts in the granuloma (Hesse et al., 2001). The presence of M2 macrophages provides a readily available supply of proline to the fibroblasts resulting in collagen synthesis. Indeed IL-4Rα LysCre mice, which are deficient in IL-4Rα specifically on macrophages and neutrophils, do not develop M2 macrophages and following *S. mansoni* infections there is endotoxemia and mortality of all infected mice (Herbert et al., 2004). It should be noted, that seminal studies showing a role for IL-13 in *S. mansoni* egg granulomatous fibrosis led to the evaluation of IL-13 as a therapeutic target in other fibrotic conditions such as asthma (Kraft, 2011; Wynn and Ramalingam, 2012). In addition to roles for cytokines chemokines are implicated in granuloma formation (Chiu et al., 2003). Chemokines such as CCL2, CCL3, CCL4, CCL7, CCL11, CCL12, and chemokine receptors CCR1, CCR2, CCR3, and CCR4 have all been shown to be associated with an exacerbated disease in animal studies and have been found at higher levels in the plasma or serum of schistosomiasis patients (Souza et al., 2008).

## *S. mansoni* Eggs and Associated Hepatotoxicity

Whilst the egg granuloma is detrimental due to the inflammation and associated fibrosis, the formation of the granuloma is essential in protecting the host from the toxins secreted by the egg. It must be stressed that in mice the development of hepatotoxicity is unique to *S. mansoni* infections, and is not seen in *S. haematobium* or *S. japonicum* (Fallon, 2000). Antigens secreted by schistosome eggs are potent inducers of the immune system and some are hepatotoxic, and if these antigens are not sequestered or neutralized the resulting inflammatory response can cause lasting damage to the host tissue (Dunne et al., 1981, 1991). As discussed above, along with T cell-dependent antibodies, the granulomatous lesions act to prevent these toxins reaching the hepatocytes (Figure 1).

Many aspects of the egg-induced immune response are mediated by glycosylated SEA (Harn et al., 1989; Hokke and Yazdanbakhsh, 2005). SEA glycoproteins collectively display a very complex set of glycans, comprising both specific schistosome glycans and molecules expressed in the mammalian host also (Jang-Lee et al., 2007). The ability of *S. mansoni* eggs to induce Th2 differentiation during infection is underscored by the observation that eggs-alone, or SEA released by the eggs through pores in the shell, is sufficient to drive Th2 polarization in naïve uninfected mice (Jankovic et al., 2004). A portion of SEA components are excreted by the schistosome egg forming the excretory/secretory (ES) fraction, while others come into contact with the host after eggs die and release their soluble contents into the surrounding tissue. Proteomic studies have shown that over a 1000 proteins can be detected in SEA, with a broad range of functions on targets located both inside (cytosolic and nuclear proteins) and outside (membrane proteins, secretory proteins) the cell (Ashton et al., 2001; Mathieson and Wilson, 2011). Distinctive glycan elements abundantly present on SEA and ES glycoconjugates are recognized by PRRs (Guo et al., 2004; Saunders et al., 2009; Ritter et al., 2010). One well characterized *Schistosoma* egg glycan is the Lewis(X)-containing lacto-*N*-fucopentaose III (LNFPIII), which has potent immunomodulatory activity (Bhargava et al., 2012; Tundup et al., 2012). In the context of defective granuloma formation leading to microvesicular steatosis during infection (Figures 1 and 2), it is interesting that LNFPIII has recently been shown to suppress liver lipogenesis and protects against hepatosteatosis (Bhargava et al., 2012; Tundup et al., 2012).

The most characterized egg secretions are derived from the highly cationic egg fraction (CEF6) of the SEA containing two important antigens, namely omega-1 (ω-1) and alpha-1 [α-1; more recently termed IPSE (IL-4-inducing principle of *S. mansoni* eggs) or *S. mansoni* chemokine binding protein (SmCKBP)]. Dunne et al. (1981) firstly formally characterized ω-1 and α-1. Immunochemical characterization of ω-1 using sera from mice and humans infected with different schistosome species clarified this antigen as specific to *S. mansoni* (Dunne et al., 1991). ω-1 is a 31 kDa monomeric glycoprotein, with a potent T2 ribonuclease (RNase) activity (Steinfelder et al., 2009) and is associated with significant hepatotoxicity (Fitzsimmons et al., 2005). In addition, transfer of antisera against ω-1 prevents hepatocyte damage in *S. mansoni*-infected T cell depleted mice confirming the hepatotoxic effects of ω-1 (Dunne et al., 1991). Recently, it has been hypothesized that ω-1 conditions mouse DCs to promote Th2 differentiation *via* a mechanism involving mannose receptors, which appear crucial for the efficient recognition and internalization of ω-1 by DCs (Everts et al., 2012). Importantly, after translocation into the cytosol, ω-1 programs DCs to drive a Th2 polarization in an RNase-dependent manner by interfering with ribosomal function and protein synthesis (Everts et al., 2012).

α-1 consists of two immunologically cross-reactive 41 and 36 kDa dimers, each of which consists of one unique and one common glycoprotein subcomponent (Dunne et al., 1991). It is particularly abundant in the sub-shell area of *S. mansoni* eggs from where it is secreted into the surrounding tissue (Schramm et al., 2003). α-1 binds Ig s and activates basophils, leading to histamine release and facilitating the production of Th2 cytokines, in particular IL-4 (Schramm et al., 2007). It has been also demonstrated that α-1 contains a functional C-terminal nuclear sequence that binds DNA leading to a potential alteration in the gene expression of the host cell (Kaur et al., 2011).

An immunomodulatory egg-antigen was identified during a screen for CKBPs in antigen extracts from *S. mansoni*. An SmCKBP was identified in SEA as a 36 kDa protein specifically secreted by live *S. mansoni* eggs (Smith et al., 2005). Binding assays showed that this egg-antigen specifically binds chemokines, such as CXCL8, CCL3, and CX_3_CL1, CCL2, and CCL5. The secretion of a CKBP by live schistosome eggs within the granuloma, suggests that this antigen may block certain chemokines to facilitate granuloma formation and alter the cellularity of the granuloma (Smith et al., 2005). As SmCKBP and α-1 are the same glycoprotein, it is an example of a molecule that may orchestrate both the cellular content and activation state of cells within the granuloma.

Collectively and paradoxically, while the eggs are the cause of pathology during *S. mansoni* infection, they also afford some protection, acting to minimize collateral tissue damage in both the liver and intestine. Therefore, even though the progression of a granuloma into a fibrotic lesion can lead to death from portal hypertension and hemorrhaging, ironically in the absence of a granulomatous response, hepatic murine schistosomiasis results in a more acute and lethal disease.

## Conclusion

Although egg-induced granulomas are detrimental to the infected host, it is clear that the inflammatory response also fulfills an important host-protective function during *S. mansoni* infection. The granuloma, rich in cells such as Th2 cells, eosinophils, and M2 macrophages, acts to protect the surrounding host tissue from the toxins released by the egg, not only by providing a physical barrier between the egg and the tissue, but also by sequestering the antigenic products secreted by the egg. This is of vital importance, particularly in the liver due to the hepatotoxicity associated with egg-antigens. However, the constant activation of the immune system over time, in particular the type 2 immune responses, results in excessive “wound healing” and invariably leads to the development of fibrotic lesions in the place of the granulomas.

While egg-associated granuloma formation clearly benefits the host, it is also associated with pathology in infected individuals. Conversely, the parasite uses the granuloma to facilitate excretion of its eggs, without killing the host, to continue the life cycle. The granuloma is thus both friend and foe during infection.

## Conflict of Interest Statement

The authors declare that the research was conducted in the absence of any commercial or financial relationships that could be construed as a potential conflict of interest.
